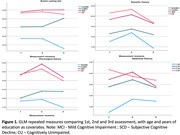# Longitudinal differences in language in middle‐aged and older people with normal cognition, subjective cognitive impairment, and mild cognitive impairment. Results of the Compostela Aging Study

**DOI:** 10.1002/alz.089078

**Published:** 2025-01-03

**Authors:** David Facal, Maria Campos‐Magdaleno, Lucía Pérez‐Blanco, Alba Felpete, Ana Nieto‐Vieites, Sabela Carme Mallo, Sonali Arora, Fátima Fernández‐Feijoo

**Affiliations:** ^1^ Deparment of Developmental and Educational Psychology, University of Santiago de Compostela, Santiago de Compostela Spain; ^2^ Applied Cognitive Neuroscience and Psychogerontology group, Health Research Institute of Santiago de Compostela (IDIS), Santiago de Compostela Spain; ^3^ Department of Developmental and Educational Psychology, University of Santiago de Compostela, Santiago de Compostela Spain

## Abstract

**Background:**

Language performance has been considered a potential screening tool in the continuum from healthy cognitive aging to cognitive impairment and dementia. Language impairment and most notably impairment in semantic verbal fluency has been shown to increase with the progress in this continuum (Liampas et al., 2023).

**Method:**

The study sample was drawn from the participants of the Compostela Aging Study who completed baseline and two follow‐up assessment, each of them about 18 months after the previous cognitive and neuropsychological evaluation (Pereiro et al., 2021). Sample included 22 persons with uni‐domain mild cognitive impairment (MCI), 20 with multi‐domain MCI, 46 with subjective cognitive decline and 73 cognitively unimpaired participants. Language performance was assessed via Boston naming test, semantic, phonemic and ideational fluency. GLM repeated measures were implemented comparing performance in the three assessments for each of the language measures as a dependent variable, and the diagnostic group as a factor. Age and years of formal education were included as covariates.

**Result:**

A significant interaction between time of assessment and group was only found for the Boston naming test (F = 1.75, *p*<0.05, η^2^ = 0,03, B‐1 = 0,53). Age, but not education, was a significant covariate for all the language measures.

**Conclusion:**

Even though language measures could be relevant to detect early changes in cognitive status, these measures has scarcely been studied in recent literature. According to these preliminary analyses of the longitudinal results of our study, the Boston naming test could be a linguistic marker of cognitive changes in late adulthood.

**References**:

Lampas, I., Folia, V., Morfakidou, R., Siokas, V., Yannakoulia, M., Sakka, P., Scarmeas, N., Hadjigeorgiou, G., Dardiotis, E., Kosmidis, M.H. (2023). Language differences among individuals with normal cognition, amnestic and non‐amnestic MCI, and Alzheimer’s disease. *Archives of Clinical Neuropsychology 38*, 525. https://doi.org/10.1093/arclin/acac080

Pereiro, A.X., Valladares‐Rodríguez, S., Felpete, A. Lojo‐Seoane, C., Campos‐Magdaleno, M., Mallo, S.C., Facal, D., Anido‐Rifón, L., Belleville, S., Juncos‐Rabadán, O. (2021). Relevance of complaint severity in predicting the progression of subjective cognitive decline and mild cognitive impairment: A machine learning approach. *Journal of Alzheimers Disease 82*, 3, 1229‐1242. https://doi.org/10.3233/JAD‐210334.